# Smooth Pursuit and Visual Occlusion: Active Inference and Oculomotor Control in Schizophrenia

**DOI:** 10.1371/journal.pone.0047502

**Published:** 2012-10-26

**Authors:** Rick A. Adams, Laurent U. Perrinet, Karl Friston

**Affiliations:** 1 The Wellcome Trust Centre for Neuroimaging, University College London, Queen Square, London, United Kingdom; 2 Institut de Neurosciences de la Timone, CNRS – Aix-Marseille University, Marseille, France; Baylor College of Medicine, United States of America

## Abstract

This paper introduces a model of oculomotor control during the smooth pursuit of occluded visual targets. This model is based upon active inference, in which subjects try to minimise their (proprioceptive) prediction error based upon posterior beliefs about the hidden causes of their (exteroceptive) sensory input. Our model appeals to a single principle – the minimisation of variational free energy – to provide Bayes optimal solutions to the smooth pursuit problem. However, it tries to accommodate the cardinal features of smooth pursuit of partially occluded targets that have been observed empirically in normal subjects and schizophrenia. Specifically, we account for the ability of normal subjects to anticipate periodic target trajectories and emit pre-emptive smooth pursuit eye movements – prior to the emergence of a target from behind an occluder. Furthermore, we show that a single deficit in the postsynaptic gain of prediction error units (encoding the precision of posterior beliefs) can account for several features of smooth pursuit in schizophrenia: namely, a reduction in motor gain and anticipatory eye movements during visual occlusion, a paradoxical improvement in tracking unpredicted deviations from target trajectories and a failure to recognise and exploit regularities in the periodic motion of visual targets. This model will form the basis of subsequent (dynamic causal) models of empirical eye tracking measurements, which we hope to validate, using psychopharmacology and studies of schizophrenia.

## Introduction

This paper is about the optimality principles that underlie oculomotor control and how one can account for particular failures in optimal control in both computational and neurobiological terms. Specifically, we consider the smooth pursuit of visual targets with periodic motion and the effect of visual occlusion on smooth pursuit eye movements. This provides a nice (a well understood and empirically studied) paradigm to model eye movements, using schemes that can be motivated from basic (Bayes optimality) principles. Furthermore, by appealing to neurobiologically plausible implementations of Bayes optimal schemes – such as active inference – one can simulate the effect of neuromodulatory deficits on optimal oculomotor behaviour and understand these deficits in computational terms. In what follows, we describe and demonstrate a model of smooth pursuit under visual occlusion and try to reproduce some common deficits seen in schizophrenia. This paper serves to introduce the model and its phenomenology. In subsequent papers, we will use this model as the basis of an observation or dynamic causal model to optimise its parameters using empirically recorded eye movements. This should allow us to test the model assumptions using psychophysical and pharmacological interventions which, if successful, may provide non-invasive measures of synaptic function mediating smooth pursuit eye movements.

The model of smooth pursuit presented below is based upon the notion of active inference. Active inference is a corollary of the principle of free energy minimisation and says that we sample sensory inputs to minimise prediction errors. Clearly, prediction errors depend upon predictions and inference on hidden states of the world causing sensory data. A crucial aspect of this inference is the proper weighting of sensory evidence and prior beliefs – in the context of uncertainty about hidden states. Operationally, this rests upon weighting prediction errors in accord with their precision. In neurobiologically plausible implementations of active inference, precision is thought to be encoded by the postsynaptic or neuromodulatory gain of neuronal populations encoding prediction errors [1]. This is important, because many psychopathologies implicate modulatory neurotransmitter systems and a putative failure of postsynaptic gain control. We will exploit this link to simulate the failures of active inference (during smooth pursuit eye movements) that are typical of schizophrenia – whose pathophysiology is thought to involve abnormalities of dopaminergic and NMDA receptor function [2].

The aim of this work was to produce a model of Bayes optimal oculomotor control that could account for the cardinal features of smooth pursuit in normal subjects and three characteristic deficits seen in schizophrenia:

- When normal subjects track a predictable target, compelling psychophysical and modelling studies suggest that they anticipate the reappearance of the target from behind an occluder. On target disappearance, eye velocity decreases to zero unless the subject expects the target to reappear, in which case – after an initial deceleration – eye velocity increases again [3]. This is taken as evidence that subjects have an internal representation of target motion that is used to provide top-down oculomotor control in the absence of visual input.
- Schizophrenics show a diminished gain in pursuit movements – from about 85 to about 75% – especially when a target is occluded: in normal subjects, at velocities of around 20deg/s, the ratio of mean eye and target velocities falls to around 60 to 70% during occlusion, while in schizophrenia it drops to 45 to 55% [4]. In other words, schizophrenics produce slower (more uncertain) pursuit movements when the target is occluded.
- Paradoxically, schizophrenics show better than normal performance in the brief period after a target unexpectedly changes direction: for around 30 ms – they change their eye velocity to match the target velocity more accurately than normal subjects [5].
- Schizophrenics fail to recognise or exploit regularities in successive repetitions of target trajectories, during smooth pursuit: normal performance on the first presentation of a target trajectory is imperfect but – after repeated presentations – normal subjects come to match it optimally, whereas schizophrenics do not [6].

In brief, we were able to explain anticipatory smooth pursuit eye movements using a hierarchical model of target motion that provided top-down (extra-retinal) predictions about hidden motion during visual occlusion. By reducing the precision of these top-down (empirical prior) beliefs, we were able to simulate the three abnormalities above; namely, a slowing of smooth pursuit during visual occlusion, a paradoxical increase in the accuracy of tracking unexpected motion and a failure to recognise regularities in target motion. In short, with a single change in an otherwise optimal scheme, we were able to explain three established signs of schizophrenia – in a neurobiologically plausible fashion.

This paper comprises five sections. In the first, we represent a brief review of empirical and theoretical studies of smooth pursuit and visual occlusion; with a special focus on findings in schizophrenia research. The second section reviews active inference from basic principles and shows how it can be implemented in the brain in terms of predictive coding. This is particularly important here, because the predictive coding formulation highlights the importance of precision weighted prediction errors and the role of postsynaptic gain or neuromodulation in optimising perceptual inference in cortical hierarchies. The third section describes our generative or forward model that produces smooth pursuit eye movements. The behaviour of this generative model, when exposed to periodic motion, is illustrated using simulations that highlight anticipatory movements during periods of visual occlusion. The fourth section revisits the simulations of normal (Bayes optimal) pursuit when the precision of hierarchical prediction errors is reduced. In the final section, we consider how subjects accumulate evidence about periodicity of target trajectories that they can use to nuance smooth pursuit, when trajectories are repeated. We conclude with a discussion of the implications of these simulation results and how the model can be used in a more pragmatic way to verify some of its assumptions and quantify its parameters using empirical data.

### Smooth pursuit eye movements and schizophrenia

This section provides a short overview of the smooth pursuit eye movement (SPEM) literature, with a particular focus on the phenomena disclosed by visual occlusion and eye movements in schizophrenia. We will use some of these key findings – at least heuristically – to motivate the form of the model used in later sections. We also appeal to studies reviewed here to motivate the potential importance of schizophrenia as a lesion-deficit model of false inference, caused by neuromodulatory failures in the encoding of precision or uncertainty.

Our aim in this and future work is to build a neurobiologically plausible computational model of an inferential process that is abnormal in both schizophrenia and those at high genetic risk. If lesioning the model – in a way that is consistent with known pathology in schizophrenia – can reproduce abnormalities of perceptual inference, then we might be able to: (i) quantify model parameters using empirical data (from both behavioural and neuroimaging studies), and (ii) interpret other abnormalities in schizophrenia as similar ‘lesions of inference’ in other brain systems.

The inferential process we have chosen to model is that of SPEM. There are several reasons motivating this choice. First, abnormal SPEM is a good candidate endophenotype for schizophrenia, with an effect size of around one [7]. Second, unlike brainstem-derived saccadic eye movement, SPEM is a cortically driven process [8], whose output is not modified downstream of the frontal eye fields [9]; it is therefore amenable to investigation by magnetoencephalography (MEG). Third, its behavioural expression can be recorded precisely and quantitatively using an eye tracker.

Attempts to quantify SPEM abnormalities in schizophrenia have used both global and specific measurements. Global measurements – e.g., qualitative measures or measures of the average distance from the eye to the target – yield the highest effect sizes, but cannot pinpoint an underlying deficit [10]. More specific measurements include *gain*: gain is defined as the ratio of eye velocity to target velocity during either the initial *open-loop* phase – in which the effects of eye movement on target fixation have not yet reached the cortex – or the *closed-loop* maintenance of pursuit. Other measurements include the numbers of catch-up saccades – that re-fixate a target – or intrusive saccades that de-fixate a target. Although most SPEM measurements are abnormal in schizophrenia, decreased maintenance gain is the specific measure with the largest effect size and narrowest confidence intervals (mean *d* −0.87+/−0.13, in [10]). This has led several authors to conclude that a fundamental problem in schizophrenia is a lag of the eye behind the target, with a compensatory increase in catch-up saccades [11], [12]. This conclusion is reinforced by data from the relatives of schizophrenics, who also show diminished maintenance gain (mean *d* −0.42), albeit without a concomitant increase in catch-up saccades [13]. Note that impaired pursuit is not the *only* fundamental problem in schizophrenia: others include reduced response inhibition, found in anticipatory and anti-saccade performance.

Why does the eye lag the target in schizophrenia? Attempts to answer this question have tested components of existing models of predictable SPEM, such as that proposed by Barnes [14]. Other models include the pioneering models of unpredictable pursuit [15], [16] and more recently neural network models implementing optimal control theory [17]. The Barnes model comprises modules for: retinal image velocity detection, gain control – that transforms the representation of target motion into oculomotor commands; efference copy of the oculomotor command – to continue pursuit during brief target occlusion; and a short-term velocity memory – that can learn and store trajectories over longer periods, allowing better anticipation of target movement. All of these functions have been shown to be abnormal in schizophrenia; namely, velocity detection [18] – subsequently attributed to problems using efference copy [19], the maintenance of target motion representation or its use in generating oculomotor commands [20], and the learning or anticipation of trajectories [6].

Investigators have tried to explain these apparently disparate problems in terms of an underlying functional abnormality. One such function – that has long been suspected to be aberrant in schizophrenia – is that of prediction: specifically, the prediction of sensory input. In one of the best validated explanations of a psychotic symptom, Frith [21] proposed that passivity experiences (delusions of control) could result from the failure of a forward model to predict accurately the sensory consequences of a motor command. He argued that if the feeling of agency for a movement depends upon the accurate prediction of its consequences – rather than the mere issuing of the motor command – this feeling could be lost if the prediction failed. Schizophrenics have been demonstrated to have deficits in predicting the sensory consequences of their actions in numerous paradigms; e.g., force-matching [22], retinal motion attribution [23], visuomotor [24] and virtual reality [25] – and in the latter three experiments, prediction deficits correlated with the strength of their passivity experiences.

Prediction deficits – or more formally, a weakened influence of prior expectations on perception and learning [26] – have also been proposed to underlie many other phenomena in schizophrenia. Examples include: decreased susceptibility to illusions such as the size-weight illusion [27] and the hollow mask illusion [28], increased susceptibility to the rubber hand illusion [29], decreased susceptibility to conditioning effects such as latent inhibition [30] and Kamin blocking [31], and numerous electrophysiological phenomena [32].

If schizophrenic prediction deficits underlie the lag of eyes behind their targets, then one would expect to see greater SPEM abnormalities in tasks with a greater predictive component. Indeed, smooth pursuit of a pseudorandom stimulus in schizophrenia is no different to that of controls': a deficit only becomes apparent once the target motion is sinusoidal, i.e. predictable [33]. Researchers have also addressed this hypothesis using paradigms in which SPEM does not depend on target motion, but on extra-retinal signals; i.e., predictions of target motion. Such paradigms involve the use of occluders or a technique called foveal stabilization, in which – unknown to the subject – eye tracker feedback is used to keep the target foveated for a brief period, which ensures that eye movement is driven purely by expectation, not by retinal slip of the image.

In normal subjects – asked to maintain pursuit during target disappearance – occlusion causes eye velocity to fall after around 200 ms until it stabilizes (at around 450 ms) at roughly half the initial velocity. This ‘residual predictive pursuit’ can be maintained for at least 4 seconds [34]. If the reappearance of the target is predictable (e.g., using a constant occluder size), eye velocity increases – after a few hundred milliseconds – back toward target velocity, although interestingly this anticipatory acceleration is not time locked to the target's reappearance [3].

Several metrics have been used to characterise the predictive element of SPEM [4]. The ‘mean predictive gain’ is the average gain during occlusion (by an occluder in the middle of a ramp). Excluding the initial deceleration period gives the ‘residual predictive gain’. An even purer measure of memory-driven prediction can be obtained by placing the occluder at the point at which a target changes direction – so that the eye's change of direction must be driven solely by past experience: in this context, the ‘peak predictive gain’ is derived from the peak eye velocity/expected target velocity during the occlusion.

There is evidence that these predictive measures are more sensitive to schizophrenic SPEM dysfunction than the popular ‘maintenance (closed-loop) gain’. Thaker and colleagues [4] showed that mean predictive gain was lower in schizophrenics, even at the low velocity of 9deg/sec, when their maintenance gain is normal. Similarly, in their study of the first degree relatives of schizophrenics and community subjects both with and without schizotypal personalities, they showed no group differences in maintenance gain, but the schizotypal relatives had significantly poorer residual predictive gain [35]. Interestingly, the relatives' peak predictive gain was also significantly poorer, irrespective of whether they were schizotypal or not. Similar results were obtained from a large community subject sample, in which *all* schizotypal individuals (disorganized subtype) had significantly lower residual predictive gain [36], whereas only the high-scoring (>2SD) disorganized schizotypal subjects had diminished maintenance gain [37].

Two other findings suggest that predictive pursuit measures something quite distinct from maintenance gain, and that this distinct predictive component could be a more specific endophenotype for schizophrenia: in both normal and schizotypal individuals, residual pursuit deficits are independent of maintenance gain; indeed, in normal subjects they were weakly anti-correlated at high speed [36], [38]. Predictive pursuit gain also has a much higher heritability (as measured in schizophrenics and their siblings) than maintenance gain – 0.9 versus 0.27 respectively – indicating that it has a much more specific genetic component [39].

The latter study demonstrates why one should not assume that the meta-analytic finding [10] that maintenance gain has a larger effect size than predictive measures in schizophrenia (0.87 with 95% confidence intervals 0.74–0.99 versus 0.35 and 0.37 with 95% confidence intervals up to 1 but both including zero) implies that it is closer to a core neurobiological deficit. In fact, the opposite is true: maintenance gain is likely to be affected by other disease-related factors, and the authors comment that its greater effect size may well be due to the disparity in the numbers of studies examining maintenance (42) versus predictive (5) gain – and the fact that the former is based on a greater proportion of the eye movement record than the latter. Indeed, Hong and colleagues showed that refining the predictive pursuit measure can substantially increase its effect size (in schizophrenic's relatives): from 0.23 (residual predictive gain) to 0.49 (peak predictive gain) to 0.87 (using foveal stabilisation) [20].

Two further potential consequences of predictive deficits in schizophrenia are important to note: the first is the finding that impaired performance of the initial ‘open-loop’ segment of smooth pursuit is not due to poor immediate processing of velocity information, but due to impaired learning of target trajectories over trials; hence control and schizophrenics perform equally badly on the first trajectory presentation, but controls subsequently learn the trajectory; i.e. they are better able to predict trajectories on the basis of past experience [6]. The second is an example of a rare scenario, in which schizophrenics perform better than controls: when there is an unexpected change in target trajectory, the former show higher maintenance gain than controls in the 120–150 ms period after the trajectory change [5]. As the authors comment, this finding supports the idea that schizophrenics – and their relatives [40] – compensate for their problems in predicting target motion by increasing their reliance on immediate sensory information. This is consistent with the finding that schizophrenics have decreased frontal (predictive) and increased occipitotemporal (sensory) activations on fMRI during SPEM compared with controls [41]. These findings relate to those of Voss and colleagues [42], who measured the predictive and retrospective binding of actions and their effects in time – in schizophrenics and controls – demonstrating that the schizophrenics showed no predictive component but an increased retrospective (reactive) component relative to controls.

The concept of the brain as a predictive coding network [43], or an inference engine, performing perceptual inference and learning using empirical Bayes [44] allows one to frame predictive pathology in schizophrenia within Bayesian models of psychosis [45]–[47]. In such models, the relative contribution of top-down prior expectations and bottom-up sensory evidence (or prediction error) to a percept is determined by their relative precisions. Decreased precision at higher levels of a predictive coding hierarchy attenuates the contribution of top-down predictions – called empirical priors – to a percept. In predictive coding schemes, this attenuates prediction errors at high levels of the hierarchy, leading to a failure of optimal prediction and greater prediction errors at the sensory level. Interestingly, abnormal prediction error responses have been demonstrated in the midbrain in both reward-related [48] and associative learning tasks [49] in schizophrenia, and these provide a compelling explanation for abnormal salience [50]. This failure of optimal prediction also explains the diminished mismatch negativity and P300 potentials (a failure to predict regularities and consequent violations) and the increased P50 auditory potential (a failure to predict the auditory input) in schizophrenia [32]. In summary, the role of precision in balancing the confidence in top-down prior beliefs, in relation to sensory evidence, is crucial for optimal inference. Functionally, both delusions and hallucinations could be regarded as instances of false inference [51]. Under predictive coding, this false inference is expressed in terms of an abnormal modulation of prediction error responses, which provides a compelling explanation for some neurophysiological abnormalities seen in schizophrenia. In the next section, we consider predictive coding in more detail and the key role of precision in active inference.

### Generalised filtering, free energy and active inference

This section sets out the basic theory used in the simulations. It introduces active inference in terms of generalised predictive coding or Bayesian filtering. We will start with a very general formulation of these schemes using the concept of variational free energy. In brief, active inference can be regarded as equipping standard Bayesian filtering schemes with classical reflex arcs that enable action to fulfil predictions about hidden states of the world. We will describe the formalism of active inference in terms of differential equations describing the dynamics of the world and internal states of the visual-oculomotor system. The neurobiological implementation of these differential equations is then interpreted in terms of predictive coding, which includes prediction errors on the motion of hidden states – such as the location of a visual target. This scheme is used in subsequent sections to simulate smooth pursuit eye movements under visual occlusion and different levels of uncertainty (precision) about hierarchical predictions.

The scheme used to model smooth pursuit eye movements in this paper has been used to model several other processes and paradigms in neuroscience (see Table 1). This active inference scheme is based upon just three assumptions:

- The brain minimises the free energy of sensory inputs defined by a generative model.
- The generative model used by the brain is hierarchical, nonlinear and dynamic.
- Neuronal firing rates encode the expected state of the world, under this model.

**Table 1:** Processes and paradigms that have been modelled using generalised filtering.

Domain	*Process or paradigm*
**Perception**	*Perceptual categorisation (bird songs)* [68]
	*Novelty and omission-related responses* [68]
	*Perceptual inference (speech)* [90]
**Sensory learning**	*Perceptual learning (mismatch negativity)* [69]
**Attention**	*Attention and the Posner paradigm* [1]
	*Attention and biased competition* [1]
**Motor control**	*Retinal stabilization and oculomotor reflexes* [61]
	*Saccadic eye movements and cued reaching* [61]
	*Motor trajectories and place cells* [91]
**Sensorimotor integration**	*Bayes-optimal sensorimotor integration* [61]
**Behaviour**	*Heuristics and dynamical systems theory* [92]
	*Goal-directed behaviour* [73]
**Action observation**	*Action observation and mirror neurons* [91]

The first assumption is the free energy principle, which leads to active inference in the embodied context of action. The free energy here is a proxy for Bayesian model evidence that is easy to compute (see Text S1). In Bayesian terms, minimising free energy means that the brain maximises the evidence for its model of sensory inputs [52]–[58]. This is the *Bayesian brain* hypothesis [59], [60]. If we also allow action to maximise model evidence we get *active inference* [61]. In this setting, desired movements are specified in terms of prior beliefs about state transitions or the motion of hidden states in the generative model. Action then realises prior beliefs (policies) by sampling sensory input to provide evidence for those beliefs.

The second assumption above is motivated by noting that the world is both dynamic and nonlinear and that hierarchical structure emerges inevitably from a separation of temporal scales [62], [63]. The final assumption is the Laplace assumption that, in terms of neural codes, leads to the *Laplace code*, which is arguably the simplest and most flexible of all neural codes [64]. See Text S2 for a motivation of the Laplace assumption from basic principles.

**Figure 1:**
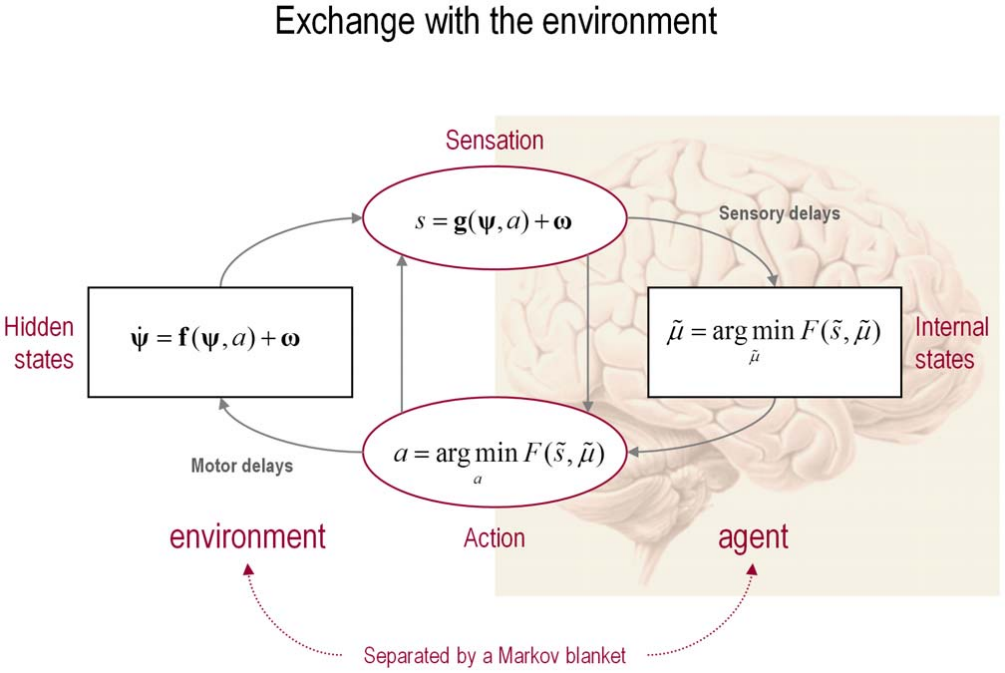
Exchange with the environment. This schematic shows the dependencies among various quantities modelling exchanges of an agent with the environment. It shows the states of the environment and the system in terms of a probabilistic dependency graph, where connections denote directed dependencies. The quantities are described within the nodes of this graph – with exemplar forms for their dependencies on other variables (see main text). Hidden (external) and internal states of the agent are separated by action and sensory states. Both action and internal states – encoding a conditional probability density function over hidden states – minimise free energy. Note that hidden states in the real world and the form of their dynamics can be different from that assumed by the generative model; this is why hidden states are in bold. See main text for further details.

Under these assumptions, action and perception can be regarded as the solutions to coupled differential equations describing the dynamics of the real world and the behaviour of an agent. These equations can be expressed in terms of action and internal states that encode conditional expectations about hidden states of the world [61]:
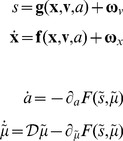
(1)See Figure 1 for a schematic summary of the implicit conditional dependencies implied by Equation (1). For clarity, real-world states are written in boldface, while internal states of the agent are in italics (for a glossary of mathematical symbols used here, see Table 2). The ∼ notation denotes variables in generalized coordinates of motion where, using the Lagrange notation for temporal derivatives: 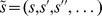 [65]. The pairs of equations are coupled because sensory states 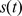 depend upon action through hidden states and causes 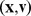, while action 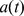 depends upon sensory states through internal states 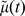. The first pair of coupled stochastic differential equations describes the dynamics of hidden states and causes in the world and how these generate sensory states. These equations are stochastic because sensory states and the motion of hidden states are subject to random fluctuations 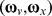. The second pair of differential equations corresponds to *action* and *perception* respectively – they constitute a gradient descent on variational free energy.

**Table 2:** Glossary of mathematical symbols.

Variable	Short description
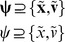	Hidden states and causes (**boldface** – real and *italic* – assumed)
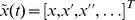	Generalised hidden states
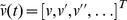	Generalised forces or causes that act on hidden states
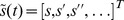	Generalised sensory states caused by hidden states
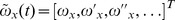	Generalised random fluctuations in the motion of hidden states
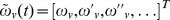	Generalised random fluctuations in hidden causes
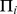	Precision (inverse covariance) of generalised random fluctuations
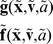	Sensory mapping and equations of motion generating sensory states
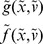	Sensory mapping and equations of motion modelling sensory states
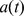	Action
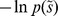	Surprise or negative log evidence of generalised sensory states
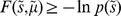	Free-energy bound on surprise
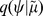	Recognition density on causes with sufficient statistics 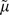
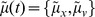	Conditional or posterior expectation of hidden states and causes
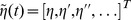	Prior expectation of generalised hidden causes
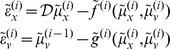	Generalised prediction error on the motion of hidden states and causes at the i-th hierarchical level

The last differential equation describing perception is known as *generalised filtering* or *predictive coding* and has the same form as standard Bayesian (Kalman-Bucy) filters – see also [43], [66]. The first term is a prediction based upon a differential operator 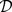 that returns the generalised motion of the conditional expectations – such that 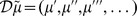. The second is an update term that ensures the changes in conditional expectations are Bayes-optimal predictions of hidden states of the world – in the sense that they maximise (the free energy bound on) Bayesian model evidence.

To perform simulations using this scheme, one simply integrates or solves Equation (1) to simulate (neuronal) dynamics that encode conditional expectations and ensuing action. Conditional expectations depend upon a generative model, which we assume has the following (hierarchical) form
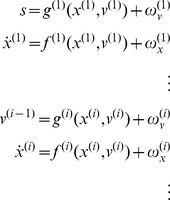
(2)This equation is just a way of writing down a generative model that specifies a probability density function over sensory inputs and hidden states and causes. This probability density is needed to define the free energy of sensory input (see Text S1): it is specified in terms of some functions and Gaussian assumptions about random fluctuations 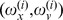 on the motion of hidden states and causes. It is these that make the model probabilistic – they play the role of sensory noise at the first level and induce uncertainty about states at higher levels. The (inverse) amplitudes of these fluctuations are quantified by their precisions 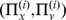.

The deterministic part of the model is specified by nonlinear functions 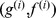 of hidden states and causes that generate dynamics and sensory consequences. Hidden causes link hierarchical levels, whereas hidden states link dynamics over time. Hidden states and causes are abstract quantities that the brain uses to explain or predict sensations – like the motion of an object in the field of view. In hierarchical models of this sort, the output of one level acts as an input to the next. This input can produce complicated convolutions with deep (hierarchical) structure. We will see examples of this later.

### Perception and predictive coding

Given the form of the generative model (Equation 2) one can write down the differential equations (Equation 1) describing neuronal dynamics in terms of prediction errors on the hidden causes and states. These errors represent the difference between conditional expectations and predicted values, under the generative model (using 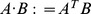 and omitting higher-order terms):
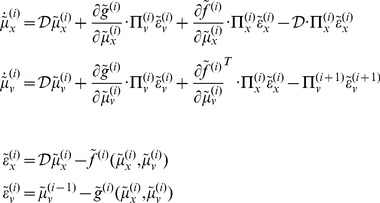
(3)
Equation (3) can be derived fairly easily by computing the free energy for the hierarchical model in Equation (2) and inserting its gradients into Equation (1). This gives a relatively simple update scheme, in which conditional expectations are driven by a mixture of prediction errors, where prediction errors are defined by the equations of the generative model.

It is difficult to overstate the generality and importance of Equation (3) – its solutions grandfather nearly every known statistical estimation scheme, under parametric assumptions about additive noise [67]. These range from ordinary least squares to advanced variational deconvolution schemes. In this form, one can see clearly the relationship between predictive coding and Kalman-Bucy filtering – changes in conditional expectations 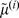 comprise a prediction (first term) plus a weighted mixture of prediction errors (remaining terms). The weights play the role of a Kalman gain matrix and are based on the gradients of the model functions and the precision of random fluctuations.

**Figure 2:**
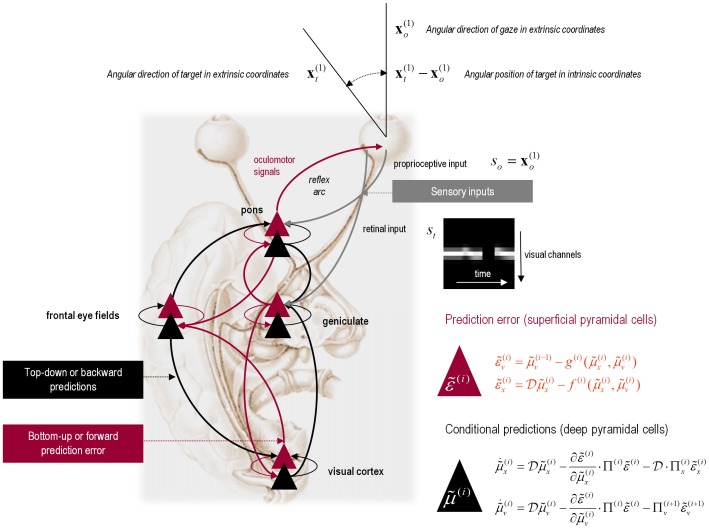
Hierarchical message passing in the visual-oculomotor system. Schematic detailing a neuronal message passing scheme (generalised Bayesian filtering or predictive coding) that optimises conditional expectations about hidden states of the world, given sensory (visual) data and the active (oculomotor) sampling of those data. This diagram shows the speculative cells of origin of forward driving connections (in red) that convey prediction error from a lower area to a higher area and the backward connections (in black) that construct predictions [70]. These predictions try to explain away prediction error in lower levels. In this scheme, the sources of forward and backward connections are superficial (red) and deep (black) pyramidal cells respectively. The equations on the right represent a generalised descent on free energy under the hierarchical model described in the main text – this can be regarded as a generalisation of predictive coding or Kalman filtering: see [67]. State-units are in black and error-units are in red. Here, we have placed different levels of some hierarchical model within the visual-oculomotor system. Visual input (illustrated in the retinal input graph) arrives in an intrinsic (retinal) frame of reference that depends upon the angular position of a stimulus and the direction of gaze. Exteroceptive input is then passed to the lateral geniculate nuclei (LGN) and to higher visual (we are merging V1–V5) and prefrontal (e.g., frontal eye fields) areas in the form of prediction errors. Crucially, proprioceptive sensations are also predicted, creating prediction errors at the level of the cranial nerve nuclei (pons). The special aspect of these proprioceptive prediction errors is that they can be resolved through classical reflex arcs – in other words, they can elicit action to change the direction of gaze and close the visual–oculomotor loop.

In neural network terms, Equation (3) says that error-units 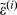 receive predictions from the same hierarchical level 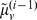 and the level above 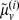. Conversely, conditional expectations (encoded by the activity of state units) are driven by prediction errors from the same level 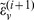 and the level below 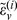. These constitute bottom-up and lateral messages that drive conditional expectations towards a better prediction to reduce the prediction error in the level below. This is the essence of recurrent message passing between hierarchical levels to suppress free energy or prediction error: see [68] for a more detailed discussion. In neurobiological implementations of this scheme, the sources of bottom-up prediction errors, in the cortex, are thought to be superficial pyramidal cells that send forward connections to higher cortical areas. Conversely, predictions are conveyed from deep pyramidal cells by backward connections, to target (polysynaptically) the superficial pyramidal cells encoding prediction error [69], [70]. Equation (3) shows how precision 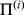 plays an important role in weighting the influence of prediction errors 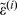 at any particular level of the hierarchy. In other words, by changing the precision on the prediction errors, we can bias inference towards sensory information or top-down (empirical) priors – empirical priors are simply beliefs encoded probabilistically that provide top-down constraints on hierarchically lower levels. Crucially, in the current context, precision corresponds to the gain of (superficial pyramidal) populations encoding prediction error and has been discussed as mediating attention and action selection [1], [71]. In later sections, we will change precision to simulate pathology of synaptic gain and consequent failures of hierarchical inference. Figure 2 provides a schematic of the proposed message passing among hierarchically deployed cortical areas.

### Action

In active inference, conditional expectations elicit behaviour by sending predictions down the hierarchy to be unpacked into proprioceptive predictions at the level of (pontine) cranial nerve nuclei and spinal-cord. These engage classical reflex arcs to suppress proprioceptive prediction errors and produce the predicted motor trajectory
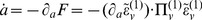
(4)The reduction of action to classical reflexes follows because the only way that action can minimize free energy is to change sensory (proprioceptive) prediction errors by changing sensory signals; cf., the equilibrium point formulation of motor control [72]. In short, active inference can be regarded as equipping a generalised predictive coding scheme with classical reflex arcs: see [61], [73] for details. The actual movements produced clearly depend upon (changing) top-down predictions that can have a rich and complex structure.

### Summary

In summary, we have derived equations for the dynamics of perception and action using a free energy formulation of adaptive (Bayes-optimal) exchanges with the world and a generative model that is both generic and biologically plausible. A technical treatment of the material above will be found in [65], which provides the details of the generalised filtering used to produce the simulations in the next section. Heuristically, these simulations simply involve integrating or solving equation (1), given a generative model in the form of equation (2). The integration scheme we use is described in Text S3 and can be considered a simulation of neuronal processing with predictive coding (equation 3) and oculomotor reflexes (equation 4) – this is active inference.

## Methods

This section introduces the generative model for smooth pursuit used to illustrate normal behaviour and, in the next section, the abnormal behaviour that results from changing the precision of prediction errors in hierarchical inference. In brief, the neuronal simulations require us to specify the equations of motion and sensory mapping from the real world and the corresponding functions that constitute a subject's generative model. To reproduce anticipatory eye movements, during visual occlusion, we require a hierarchical generative model that represents hidden motion. For simplicity, we will only consider (horizontal) motion in one dimension and ignore vertical motion.

### Oculomotor following model

The generative model for smooth pursuit eye movements used here is very simple and is based upon the prior belief that the centre of gaze and target are attracted to a common (fictive) attractor in visual space. The process generating sensory inputs is however much simpler and can be expressed as follows:
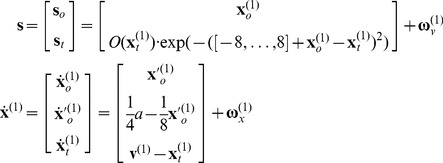
(5)This pair of equations corresponds to the noisy sensory mapping from hidden states and the equations of motion for those states in the real world. The real world provides sensory input in two modalities (see Figure 2): proprioceptive input from cranial nerve nuclei reports the (horizontal) angular displacement of the eye 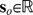 and corresponds to the centre of gaze in extrinsic coordinates 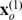. Exteroceptive (retinal) input reports the angular position of a target in a retinal (intrinsic) frame of reference 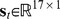. This input models the response of 17 visual channels, each equipped with a Gaussian receptive field with a width of one angular unit and deployed at intervals of one angular unit – about 2° of visual angle. This input can be occluded by a function of target location 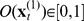, which turns values between zero and one, such that whenever the target location 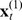 is behind the occluder, retinal input 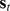 falls to zero. The response of each visual channel depends upon the distance of the target from the centre of gaze. This is just the difference between the oculomotor angle and target location in an extrinsic frame of reference: 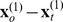.

The hidden states of this model comprise the oculomotor states – oculomotor angle and velocity
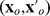
and the target location 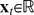. Oculomotor velocity is driven by action and decays to zero with a time constant of eight time bins or 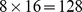 milliseconds. This means the action applies forces to the oculomotor plant, which responds with a degree of viscosity. The target location is perturbed by the hidden cause 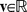 that describes the location to which the target is drawn (a sinusoid), with a time constant of one time bin or 16 ms. More specifically, changes in target location 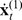 are driven by the difference between an attracting position 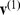 and its current location 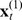. In this paper, the random fluctuations on sensory input and the motion of hidden states were virtually absent, with a log precision of 16. In other words, the random fluctuations have a variance of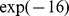. This completes our description of the process of generating sensory information; in which hidden causes produce horizontal motion of a target location and action forces oculomotor states. Target location and oculomotor states are combined to produce sensory information about the target in an intrinsic (retinal) frame of reference over an array of sensory channels.

The generative model has a similar form to equation (5) but with two important exceptions: there is no action and the motion of the hidden oculomotor states is driven by the same hidden cause that moves the target. In other words, the agent believes that its gaze is attracted (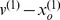) to the same fictive point in visual space that is attracting the target (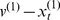). Second, the generative model is equipped with a deeper (hierarchical) structure that can represent periodic trajectories in the hidden cause of target motion:
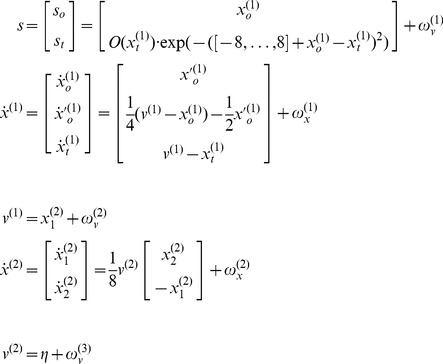
(6)


These equations constitute the probabilistic model of how sensations are generated in the form of Equation 2. This model defines the free energy in Equation 1 – and specifies behaviour under active inference. The sensory mapping of the generative model is exactly the same as above. The equations of motion for the hidden oculomotor states and target location are very similar; apart from the fact that oculomotor velocity is now driven by the displacement between the oculomotor angle and hidden cause. However, in the generative model hidden causes are informed by the dynamics of hidden states at a second level 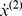. These hidden states model underlying periodic dynamics using a simple periodic attractor 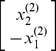 that produces sinusoidal fluctuations of any amplitude and a frequency that is determined by a second level hidden cause 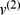
with a prior expectation of 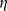. This prior expectation corresponds to beliefs about the frequency of periodic motion of the target. In the simulations below, we used a fixed prior, which was set to the correct frequency with a wavelength of 56 (simulation with occluders) or 32 (remaining simulations) time bins. The log precisions on the random fluctuations in the generative model were three at the first (sensory) level and minus one at the higher level, unless stated otherwise. This means that the agent is more confident about its sensory input than it is about how that sensory input will evolve, as determined by the (motion of) hidden states and causes. This situation is equivalent to that of an experimental subject viewing a pursuit paradigm for the very first time: [s]he can see the target clearly but is uncertain of its amplitude and frequency until it has completed at least one cycle. In the last section, we will reduce the precision on the hidden causes at the second level to simulate inference on the periodicity of the target trajectory on repeated exposure.

Having specified the generative process and model, we can now solve the active inference scheme in Equation 1 and examine its behaviour. This generative model produces smooth pursuit eye movements because it embodies prior beliefs that its gaze and the target are attracted by the same hidden causes. This smooth pursuit rests on conditional expectations about the target location in extrinsic coordinates and the state of the oculomotor plant, where target location is driven by hidden causes that also have to be inferred.

## Results

### Simulating normal subjects

**Figure 3:**
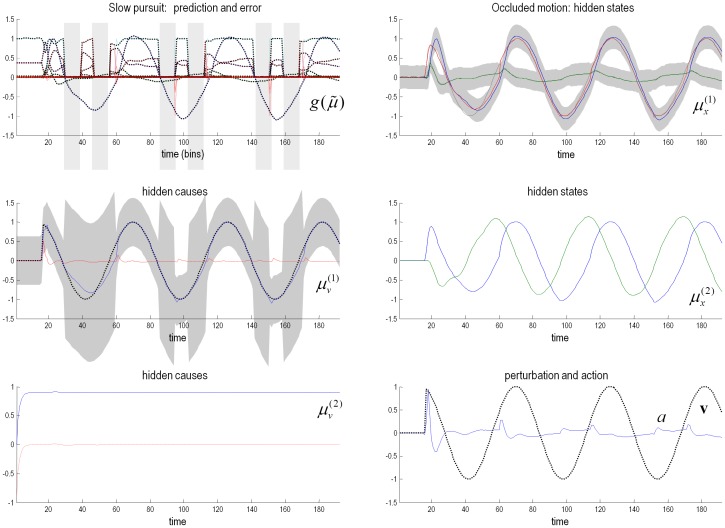
Simulation of smooth pursuit of a partially occluded target. This figure reports the conditional estimates of hidden states and causes during the simulation of smooth pursuit eye movements, using horizontal sinusoidal target motion with a period of 56 time bins – starting at 16 time bins. All times are measured in 16 ms time bins. The target was occluded whenever it passed behind an occluder at a leftward displacement of 0.1° to 1.8° of visual angle. The upper left panel shows the predicted sensory input (coloured lines) and sensory prediction errors (dotted red lines) along with the true sensory input (broken black lines). The different coloured lines correspond to photoreceptor activity over the array of (17) sensory inputs. The proprioceptive sensations (blue lines) reflect a veridical smooth pursuit, even during occlusion, indicated by the light grey bars. These sensory predictions are based upon the conditional expectations of hidden oculomotor (blue line) and target (red line) angular displacements shown on the upper right. The grey regions correspond to 90% Bayesian confidence intervals and the broken black lines show the true values. The hidden cause of these displacements (broken black line) is shown with its conditional expectation (blue line) in the middle left panel where the prediction error on this hidden cause shown as a dotted red line. Note the increase in uncertainty about this hidden cause during the periods of occlusion; however, this uncertainty is moderated because the hidden cause is informed by the motion of hidden states at the second level – shown on the middle right. These show the anticipated periodic dynamics of appropriate amplitude to minimise prediction errors at lower levels in the hierarchy. The period of these dynamics is fixed by the hidden cause at the second level, as shown on the lower left – where the conditional expectation (blue line) reaches its prior expectation almost immediately. The true cause and action (Equation 5) are shown on the lower right. The action (blue line) is responsible for oculomotor displacements and is driven by proprioceptive prediction errors.

Figure 3 reports the conditional expectations about hidden states and causes during the simulation of smooth pursuit eye movements, using horizontal sinusoidal target motion with a period of 56 time bins – starting at 16 time bins. Crucially, the target was occluded whenever it passed behind an occluder at a leftward displacement of 0.1° to 1.8° of visual angle.

**Figure 4:**
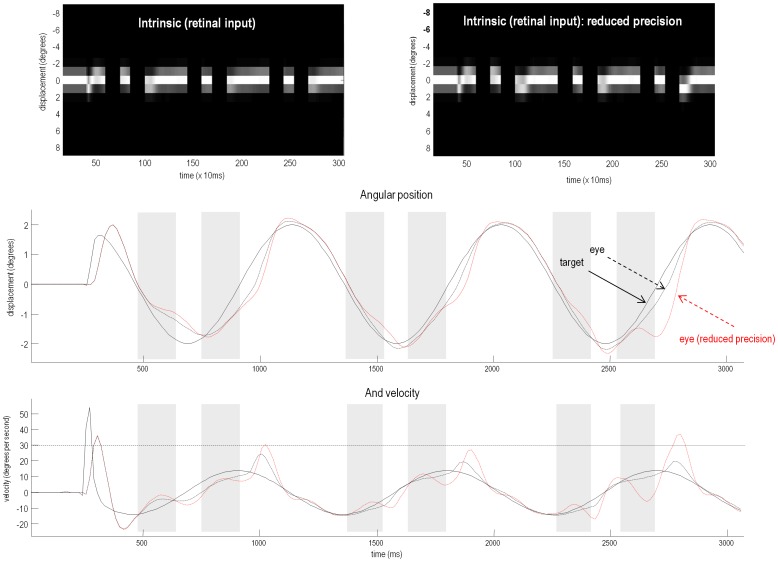
Smooth pursuit of a partially occluded target with and without high-level precision. The panels of this figure show the results of the previous in a different format: the upper left panel shows the responses of each of the (17) photoreceptors in image format as a function of peristimulus time. This shows the small fluctuations in signal that are due to imperfect pursuit and consequent retinal slip at the onset of target motion. Later, during periods of occlusion, the sensory input disappears. The lower panels show the angular displacement (top) and velocity (bottom) of the target (solid lines) and eye (broken lines) as a function of peristimulus time. The grey area corresponds to the period of visual occlusion. The equivalent results – when the precisions of prediction errors on the motion of hidden states at the second level were reduced from a log precision of −1 to −1.25 – are shown in the upper right panel and as red broken lines in the lower panels. The broken horizontal line in the lower panel corresponds to an angular velocity (30°/s) at which the eye movement could be considered saccadic. Please refer to the main text for a detailed description.

The upper left panel shows the predicted sensory input (coloured lines) and sensory prediction errors (dotted red lines) along with the true values (broken black lines – which are almost superimposed). Here, we see fluctuations in the predicted sensory input during smooth pursuit where, crucially, these inputs fall to zero during periods of occlusion (these sensory fluctuations are shown in image format in Figure 4). The proprioceptive sensations (blue lines) reflect a veridical smooth pursuit, even during occlusion. These sensory predictions are based upon the conditional expectations 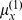 of hidden oculomotor (blue line) and target (red line) angular displacements shown on the upper right. The grey regions correspond to 90% Bayesian confidence intervals and the broken lines show the true values. One can see clearly the target motion that elicits pursuit responses that follow with a short delay of about two time bins (about 32 ms). The hidden cause of these displacements is shown (broken black line) with its conditional expectation 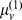 (blue line) on the middle left. Note the profound increase in uncertainty about this hidden cause during the periods of occlusion; however, this uncertainty not complete, because the hidden cause is informed by the motion of hidden states at the second level – shown on the middle right. These show the anticipated periodic dynamics of appropriate amplitude to minimise prediction errors at lower levels in the hierarchy. The period of these dynamics is fixed by a hidden cause at the second level, as shown on the lower left. The true cause and action are shown on the lower right. The action (blue line) is responsible for oculomotor displacements and is driven by proprioceptive prediction errors. For our purposes, these simulations can be regarded as Bayes optimal solutions to the smooth pursuit problem.

Figure 4 shows the same results in a different format: the top left panel shows the responses of sensory channels in image format as a function of peristimulus time. This shows the small fluctuations in signal that are due to imperfect pursuit and consequent retinal slip at the onset of target motion. Later, during periods of occlusion, the sensory input disappears. The lower panels show the angular displacement (top) and velocity (bottom) of the target (solid lines) and eye (broken black lines) as a function of peristimulus time (we will consider the red lines later). From the angular displacement trace, one can see that after some initial uncertainty about the path of the target (its speed and amplitude), the eye tracks the target fairly accurately.

These results illustrate two important aspects of pursuit behind occluders: initial effects and anticipatory effects. The first time the target passes behind the occluder, the eye tracks its location quite well to begin with, but after around 100 ms the eye velocity drops significantly and it lags the target. When it emerges at 900 ms the eye must then make a saccade-like movement to catch up with the target (although not as fast as a real saccade – taken to exceed at least 30° per second [74], as indicated by the broken horizontal line). Aside from the latency and speed of corrective saccades, which are not the focus of our modelling, this sequence is typical of normal subjects' pursuit behind occluders: e.g. see Figure 1 in [38].

On the second and third cycles of target occlusion, the eye's tracking is much better, because the agent has inferred the hidden target motion – as its conditional expectations about hidden states become entrained by its sensations. The anticipation of the target motion is so good that it only lags the target when the latter is accelerating (at 1800 ms and 2700 ms). The improved tracking of an occluded target when its reappearance is anticipated is also a well-documented finding [3]; in some cases, pursuit velocity behind the occluder does not drop at all, e.g. Figure 8 in [75].

### Summary

In summary, to account for anticipatory pursuit movements that are not apparent in target motion, one needs to equip generative models with a hierarchical structure that can accommodate latent dynamics that may or may not be expressed at the sensory level. Hierarchical extensions of this sort emphasise the distinction between visual motion processing and oculomotor control based purely upon retinal and proprioceptive input – they emphasise extra-retinal processing that is informed by prior experience and beliefs about the latent causes of visual input. These beliefs and associated inference are disclosed nicely by visual occlusion. In the next section, we look at simulated lesions to this Bayes optimal pursuit behaviour as a metaphor for the deficits seen in schizophrenia.

### Simulating psychopathology

In this section, we make one simple change to the generative model and repeat the simulations of previous section. The putative deficit in schizophrenia – reduced high-level precision – can be modelled by reducing the precision on the prediction errors at the highest level of the hierarchy. One can see how this affects conditional predictions in the first equality in equation (3) – see also Figure 2: lowering the precision 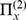 reduces the contribution of prediction errors to the conditional expectations modelling (hidden) periodic motion of the target. This results in a slowing of the (empirical prior beliefs about the) target trajectory, as confidence in the prediction errors on its motion falls. Normally, this would place more emphasis on bottom-up prediction errors to guide the trajectory; however, during occlusion these are simply absent and, in principle, we should see the behavioural effect of the ensuing loss of certainty or precision.

To model this deficit, we introduced a small reduction in the log precision of prediction error on the motion of hidden states at the second level of the generative model, from −1 to −1.25. Neurobiologically, this corresponds to a reduction in the postsynaptic gain of superficial pyramidal cells encoding prediction error in cortical areas responsible for representing high-level statistical regularities in target motion. This reduction in gain in schizophrenia may involve interactions between classical modulatory neurotransmitter systems and NMDA receptor function (see the Discussion). To examine the effects of the simulated lesion on smooth pursuit during visual occlusion, we repeated the above simulation:

Figure 4 shows the resulting sensory sampling (upper right panel) and underlying angular positions and velocities of the target and eye (red broken lines) in the lower top and bottom panels respectively. Comparison with the corresponding results under normal precision (black broken lines) shows some typical properties of schizophrenic pursuit. First, the reduced precision trace is disproportionately affected by target occlusion: at the end of occlusion, the lag behind the target is much greater than the normal precision trace on four out of six occasions: including those in which the target is actually decelerating. This is despite the fact that when the target is visible and pursuit is stabilized, the ‘schizophrenic’ tracking is no different to that of the normal eye (1200–1400 ms and 2000–2200 ms). This echoes empirical findings in schizophrenic pursuit at modest speeds (see Table 2 in [4]). Second, the reduced precision trace is more inaccurate on the third cycle than the first: it shows much less anticipatory behaviour than the normal trace. Indeed, it lags so much just prior to 2700 ms that it has to make a catch-up saccade when the target re-emerges (note the pathological catch up saccade exceeds 30° per second). We shall return to this precision-dependent difference in learning in our final simulation.

Overall, these results are consistent with findings in schizophrenia that suggest an impaired ability to maintain veridical pursuit eye movements in the absence of visual information. Furthermore, they suggest that the computational mechanism that underlies this failure rests on a failure to assign precision or certainty to (empirical) prior beliefs about hidden trajectories.

**Figure 5:**
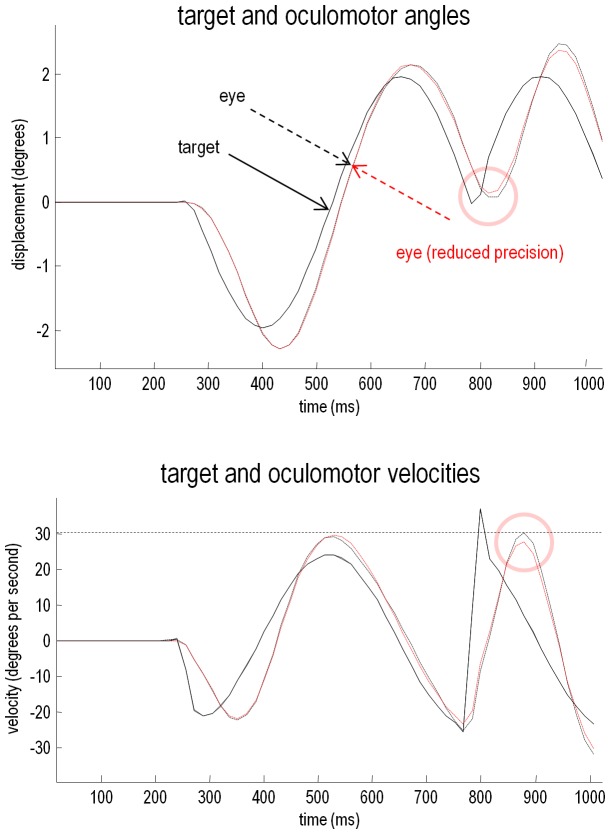
Smooth pursuit with an unexpected trajectory change, with and without high-level precision. This figure reports the results of simulations using an occluded periodic motion with a reversal in the direction of the trajectory at the beginning of the second cycle (plain black line). The broken traces in black correspond to normal pursuit and the broken traces in red show the performance under reduced precision. Although the effect is small, the low precision simulation shows more accurate pursuit performance, both in terms of the displacement between the target and centre of gaze and in terms of a slight reduction in the peak velocity during the compensatory eye movement (pink circles).

Perhaps a relative loss of certainty about top-down predictions could also explain the ability of schizophrenics to respond to unpredicted changes in direction of the target. To explore this possibility, we removed the occluder, decreased the target period to 32 time bins, and introduced an unexpected reversal in the motion of the target – at the beginning of the second cycle of motion (at around 780 ms). We then repeated the simulations using a normal generative model and the generative model with a second level precision deficit of −4 (a greater precision deficit is required to demonstrate effects when occluders are absent, because sensory precision is relatively high). The results of the simulations are shown in Figure 5, in terms of the angular displacements and velocities shown in the previous figure. The traces in black correspond to normal pursuit and the traces in red show the performance under reduced precisions. Although the effect is small (as it is in real subjects [5]), the schizophrenic simulation (red lines) shows more accurate pursuit performance, both in terms of the displacement between the target and centre of gaze and in terms of a slight reduction in the peak velocity during the compensatory eye movement – a movement that is nearly fast enough to be a saccade. These differences are highlighted by pink circles.

### Summary

In summary, a single manipulation – that has some construct validity, in relation to the pathophysiology of schizophrenia – can account for both impaired smooth pursuit eye movements during occlusion and the paradoxical improvement of responses to unpredictable changes in target direction. This dissociation makes perfect sense from the point of view of the computational anatomy we have modelled here – reducing synaptic gain (precision) at high levels of a hierarchical Bayesian inference or predictive coding scheme reduces confidence in predictions that impairs performance when these predictions are needed (during occlusion) and that improves performance when they are not (during unpredicted motion). In the simulations so far, we have assumed that the top-down predictions are veridical and that all the simulated subjects have properly inferred that the period of sinusoidal motion. In the final section, we look at how these beliefs are acquired:

### Acquiring prior beliefs

In this section, we briefly show that this Bayes optimal scheme can easily infer the dynamics of target motion through optimising its conditional expectation about the frequency of periodic motion. This can be regarded as an experience-dependent accumulation of evidence about the periodicity of target movement, during repeated exposure to the trajectory. This is a fairly difficult problem to solve, because active inference is actually changing the sensory samples at a timescale that is fairly close to the periodicity that needs to be inferred. However, if we accumulate information sufficiently slowly – by placing appropriately informative priors on the hidden causes at the second level – then we can use predictive coding to establish posterior beliefs about statistical regularities in target motion that can then be used as prior beliefs for subsequent trials.

**Figure 6:**
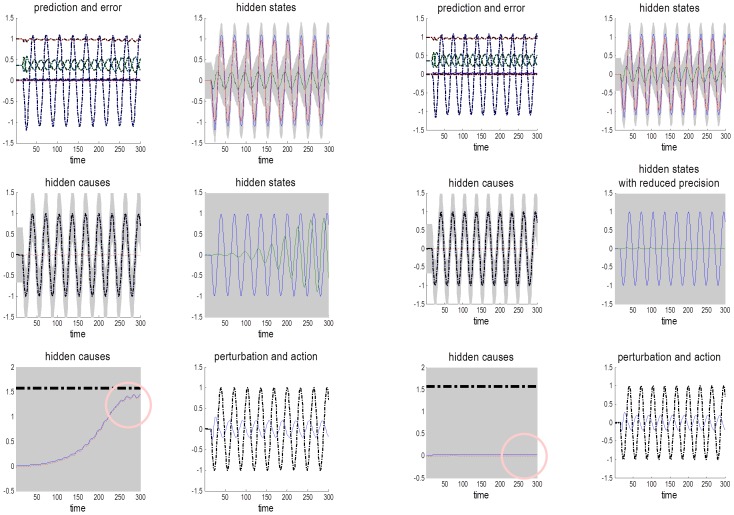
Failure to infer high-level causes when precision is low. This figure shows the results of simulating numerous cycles of periodic motion starting with a prior expectation about its frequency of zero. The panels on the left show the results of a simulation with normal precision, while the right panels present the equivalent results under lower precision. These panels use the same format as Figure 3, including time which is measured in 16 ms bins. The key result here is a failure to infer the true level (broken black lines in the lower left panels) of the hidden cause at the highest level (blue lines) when precision is low (pink circles).

To simulate this experience-dependent inference, we simply repeated the simulation of periodic motion in the absence of an occluder. To model a subject who anticipated sinusoidal motion but had no expectations about its frequency, we increased the log precision on the (second level) hidden states encoding the sinusoidal motion (from minus one to three), and reduced the log precision on the (second level) hidden cause encoding the frequency of periodic motion (from minus one to minus four), with a prior expectation of zero. The left-hand panels of Figure 6 show the results of this simulation using same format as Figure 4. It can be seen that pursuit performance is virtually the same as it was under visual occlusion. The key difference here is that the hidden states at the second level only attain the correct amplitude after nine cycles of motion. This is accompanied by a slow rise in the hidden causes at the third level (blue line) to the true level (broken black line) shown in the pink circle on the lower left – this is the inferred frequency of periodic motion. This slow rise reflects the evidence accumulation and optimisation of the posterior or conditional expectation about the periodicity of motion as more and more sensory evidence becomes available. Note that there is no discernible improvement in performance – afforded by recognising periodic motion – because the target is visible at all times and provides precise visual information. However, as we have seen the previous section, a failure to properly infer periodic motion can produce profound deficits during visual occlusion.

The key question now is whether the 3 log unit deficit in precision used in the previous section to reproduce the behavioural deficits seen in schizophrenia, also accounts for a failure to infer or recognise periodic target motion. As noted above, schizophrenics seem to have particular difficulty in recognising and exploiting statistical regularities with consequent failures of SPEM relative to control subjects after, and only after, exposure to repeated movement trajectories [6]. The right-hand panels of Figure 6 show the results of the same simulation of experience-dependent inference reported in the left panels but using the generative model with reduced precision. Crucially, this simulated lesion completely abolishes the evidence accumulation and consequent inference about periodic motion (pink circle on the right).

### Summary

There is some evidence to suggest that schizophrenics are unable to recognise or make inferences about target trajectories, in the context of smooth pursuit eye movements. The simulation results in this section suggest that these inferences are confounded by the same reduction in precision used to simulate schizophrenic pathophysiology in the previous section. The mechanism here is quite straightforward – Bayesian updates to the hidden causes at the third level are mediated by precise prediction errors from the second. Reducing the precision of these prediction errors subverts this inference by reducing the potency of bottom up prediction errors and the rate of evidence accumulation.

## Discussion

In this paper, we have considered optimal oculomotor control in the context of smooth pursuit eye movements and visual occlusion. In particular, we have taken a Bayesian perspective on optimality and have simulated various aspects of smooth pursuit using active inference. Active inference depends upon a generative model of stimulus trajectories and their active sampling through eye movement. This requires a careful consideration of the generative models that might be embodied by the visual-oculomotor system and the sorts of behaviours one would expect to see under these models. We hope to have shown that the sorts of anticipatory eye movements seen in visual occlusion paradigms can be reproduced using simulations of active inference with hierarchical models. Crucially, these models enable hidden trajectories to be represented and updated dynamically during periods of visual occlusion – and thereby inform anticipatory eye movements. We considered how one might model the pathophysiology of disorders like schizophrenia and account for the particular deficits shown by schizophrenics in terms of smooth pursuit eye movements. In brief, we have seen that a single change – a reduction in the precision or certainty with which high-level beliefs about target motion are held – provides a unifying explanation for performance deficits and paradoxical advantages – and a failure to infer contextual constraints or regularities that would otherwise improve performance.

Crucially, this single change is not implausible, given current understanding about the synaptic pathologies implicated in schizophrenia. We have described elsewhere how precision is encoded by synaptic gain of prediction error units in neuronal models of predictive coding [1], [71]. This suggests that a reduction in high-level precision could be associated with abnormalities of the synaptic gain of superficial pyramidal cells in higher cortical areas, such as prefrontal cortex (PFC). Abnormalities in two crucial determinants of synaptic gain in the PFC have long been implicated in schizophrenia: dopaminergic activity [76] and NMDA receptor function [45]. Dopaminergic and NMDA receptors have complex and interacting roles in prefrontal cortex: D1 receptor activation potentiates the slowly decaying and voltage-dependent NMDA receptor conductance, whereas D2 receptors have the opposite effect – and a large literature shows how the balance of D1 and D2 receptor activity could affect the stability of attractor networks subserving working memory in PFC; e.g. [77]–[80]. These biophysically informed models have shown how D1 activity can reinforce currently active cell assemblies whilst inhibiting the formation of new ones, and conversely D2 activity makes cell assemblies more flexible but much more unstable and vulnerable to noise. This is precisely the effect of reducing precision in the simulations above – rendering high-level dynamics less stable and susceptible to other sources of prediction error.

Building on the work of Weinberger and Goldman-Rakic [81], [82], another literature has evolved in parallel, modelling how abnormalities of synaptic gain in this system could account for many symptoms of schizophrenia. For example, Braver and colleagues used a connectionist model to show how a noisy dopamine signal could reproduce typical schizophrenic impairments on the Continuous Performance Task (CPT) [83]. They established this by increasing the variability of the gain with which cue inputs drive PFC context units – that maintain a short-term memory of the preceding cue for comparison with the current cue. Durstewitz and Seamans point out that most accounts of psychosis implicate higher D2 activity and hence a fragile and dysfunctional working memory and the other dysexecutive problems associated with the ‘disorganized’ syndrome [84]; although they add that higher D1 activity – secondary to D1 receptor upregulation due to chronic hypodopaminergia in PFC [85] could cause perseveration and disengagement from motivational processes characteristic of the ‘negative’ syndrome. Lastly, Rolls and colleagues used a dynamical-systems framework to show how NMDA receptor hypofunction could cause unstable attractor networks in different areas of PFC, accounting for both cognitive symptoms (in dorsolateral PFC) and negative symptoms (in orbitofrontal or anterior cingulate cortex) [86].

Clearly, there are many aspects of oculomotor control and schizophrenic pathophysiology we have ignored in this theoretical work. For example, we have not addressed the general reduction in oculomotor gain seen in schizophrenia prior to – or in the absence of – occlusion. Non-specific effects of this sort can be reproduced fairly easily, by reducing the precision at lower levels of the hierarchy (results not shown). We will pursue this in future work using visual targets that are degraded with high levels of sensory noise. Here, we have chosen to focus on the profound and specific deficits disclosed by visual occlusion. Another outstanding area is the relationship between our active inference scheme and previous models of oculomotor control. There is a slight disconnect between active inference and classical models based upon optimal control theory (e.g. [17]). This is because classical models rely upon a cost or value function to specify optimal trajectories. Active inference does not fall into this class of models and does not require (user-specified) cost functions. Having said this, there are some formal similarities between the optimal control models and active inference – most notably the use of prediction errors and state estimation. See [87] for a fuller discussion.

To conclude, we hope to have shown that reducing the precision at high levels in a neurobiologically plausible hierarchical inference scheme can reproduce some of the key schizophrenic abnormalities of SPEM. We have demonstrated elsewhere (see Table 1) that this model of brain function (based on generalised filtering) is plausible and accounts for a wide variety of neuronal processes and electrophysiological data. We argued that reducing the high-level precision in this model – and hence its ability to specify high-level predictions of the sensorium – is a realistic model of schizophrenic pathology for both conceptual reasons (outlined in the second section) and for the pathophysiological reasons outlined above. The former include the close parallels between this model and other theories of psychosis based on failures of prediction; e.g., Frith's account of passivity, reduced susceptibility to illusions, differences in conditioning performance and electrophysiological potentials and more recent Bayesian accounts; the latter include the dopaminergic and NMDA receptor mediated failures of prefrontal synaptic gain, which underpin many other theories of schizophrenic symptoms. The associated functional reduction in high-level precision – during hierarchical inference – reproduced various characteristic schizophrenic SPEM abnormalities: the reduction of gain during target occlusion, the poor learning of target trajectories, and the slightly improved tracking of unexpected changes in trajectory.

Further challenges remain. Among them is to use this model as the basis for a dynamic causal model [88] of MEG signals and simultaneous eye movement data from normal subjects performing smooth pursuit in the presence of occluders. If possible, we shall also attempt to model the data generated by schizophrenics and normal subjects undergoing pharmacological manipulations, and – from the consequent changes in model parameters – we ought to be able to make specific inferences about how synaptic function differs in schizophrenia and how this impacts upon both connectivity between brain regions [47] and the process of inference itself [89].

## Supporting Information

Text S1 **Variational free energy.**
(DOCX)

Text S2 **The maximum entropy principle and the Laplace assumption.**
(DOCX)

Text S3 **Integrating or solving active inference schemes using generalised descents.**
(DOCX)
