# Supplementary material for: Smooth Pursuit and Visual Occlusion: Active Inference and Oculomotor Control in Schizophrenia
Source: PLoS One. 2012 Oct 26;7(10):e47502. doi: 10.1371/journal.pone.0047502 (PMC3482214; doi:10.1371/journal.pone.0047502)
Supplement: Text S1 — Variational free energy. (DOCX) [file pone.0047502.s001.docx]

Text S1

*Variational free energy*

Here, we derive various formations of free-energy and show they relate to each other. We start with the quantity we want to bound and implicitly minimise – namely, surprise or the negative log-evidence associated with sensory states that have been caused by some unknown quantities

S1.1

We now simply add a non-negative cross-entropy or divergence between some arbitrary (conditional) density and the posterior density to create a free-energy bound on surprise

S1.2

The cross entropy term is non-negative by Gibb’s inequality. Because surprise depends only on sensory states, we can bring it inside the integral and use to show free-energy is a Gibb’s energy expected under the conditional density minus the entropy of the conditional density

S1.3

This is a useful formulation because it can be evaluated in a relatively straightforward way given a probabilistic generative model .
