# Supplementary material for: Smooth Pursuit and Visual Occlusion: Active Inference and Oculomotor Control in Schizophrenia
Source: PLoS One. 2012 Oct 26;7(10):e47502. doi: 10.1371/journal.pone.0047502 (PMC3482214; doi:10.1371/journal.pone.0047502)
Supplement: Text S2 — The maximum entropy principle and the Laplace assumption. (DOCX) [file pone.0047502.s002.docx]

Text S2

*The maximum entropy principle and the Laplace assumption*

If we admit an encoding of the conditional density up to second order moments, then the maximum entropy principle [1], implicit in the definition of free energy above, requires to be Gaussian. This is because a Gaussian density has the maximum entropy of all forms that can be specified with two moments. Assuming a Gaussian form is known as the *Laplace assumption* and enables us to express the entropy of the conditional density in terms of its first moment or expectation. This follows because we can minimise free energy with respect to the conditional covariance as follows:

S2.1

Here, the conditional precision is the inverse of the conditional covariance . In short, free energy is a function of generalised conditional expectations and sensory states.

1. Jaynes E (1957) Information Theory and Statistical Mechanics. Physical Review Online Archive (Prola) 106: 620–630.
