# Supplementary material for: Smooth Pursuit and Visual Occlusion: Active Inference and Oculomotor Control in Schizophrenia
Source: PLoS One. 2012 Oct 26;7(10):e47502. doi: 10.1371/journal.pone.0047502 (PMC3482214; doi:10.1371/journal.pone.0047502)
Supplement: Text S3 — Integrating or solving active inference schemes using generalised descents. (DOCX) [file pone.0047502.s003.docx]

Text S3

*Integrating or solving active inference schemes using generalised descents.*

Given a generative model or its associated Gibbs energy function, one can now simulate active inference by solving the following set of ordinary differential equations for a system that includes generalised real-world states and internal states of the agent mediating (delayed) action and perception:

 S3.1

Generalised action is approximated using discrete values of $a(t)$ from the past. Note that we have included a prior expectation of hidden causes to complete the agent’s generative model of its world. Integrating or solving Equation S3.1 corresponds to simulating active inference. The updates of the collective states over time steps of use a local linearization scheme [1]:

 S3.2

Details about how to compute the gradients and curvatures pertaining to the conditional expectations can be found in [2]. These are generally cast in terms of prediction errors using straightforward linear algebra. Because action can only affect free-energy through the sensory states, its dynamics are prescribed by the following gradients and curvatures:

 S3.3

The partial derivative of the sensory states with respect to action is specified by the generative process. In biologically plausible instances of this scheme, this derivative would have to be computed on the basis of a mapping from action to sensory consequences. It is generally assumed that agents are equipped with epigenetically, because it has a simple form. For example, contracting a muscle fibre elicits a proprioceptive stretch signal in a one-to-one fashion. The precision matrix in Equation S2.5 is specified such that only proprioceptive prediction errors with these simple forms have nonzero precision. This can be regarded as the motor gain in response to proprioceptive prediction errors.

Equation S3.2 may look complicated but can be evaluated automatically using numerical derivatives for any given generative model. All the simulations in this paper used just one routine - **spm_ADEM.m** - available as part of the SPM software (<http://www.fil.ion.ucl.ac.uk/spm>).

1. Ozaki T (1992) A bridge between nonlinear time series models and nonlinear stochastic dynamical systems: a local linearization approach. Statistica Sinica 2: 113–135.

2. Friston K, Stephan K, Li B, Daunizeau J (2010) Generalised Filtering. Mathematical Problems in Engineering 2010: 1–35. doi:10.1155/2010/621670.
